# Variability of cardioinhibition in vasovagal syncope: differences between subgroups during cardioinhibition and beyond

**DOI:** 10.1007/s10286-023-00991-5

**Published:** 2023-10-24

**Authors:** Ineke A. van Rossum, Frederik J. de Lange, David G. Benditt, Erik W. van Zwet, Marc van Houwelingen, Roland D. Thijs, J. Gert van Dijk

**Affiliations:** 1 Department of Neurology, Leiden University Medical Centre, PO Box 9600, 2300 Leiden, The Netherlands; 2 Heart Centre, Department of Clinical and Experimental Cardiology, Amsterdam UMC, University of Amsterdam, Amsterdam Cardiovascular Sciences, Amsterdam, The Netherlands; 3 Cardiac Arrhythmia Center, Cardiovascular Medicine, University of Minnesota, Minneapolis, MN USA; 4 Department of Medical Statistics, Leiden University Medical Centre, Leiden, The Netherlands; 5 Department of Experimental Cardiology, Erasmus Medical Centre, Rotterdam, The Netherlands; 6 Stichting Epilepsie Instellingen Nederland (SEIN), Heemstede, The Netherlands

**Keywords:** Vasovagal syncope, Reflex syncope, Cardioinhibition, Tilt-table testing

## Abstract

**Purpose:**

We compared hemodynamic parameters between subjects with marked, intermediate and minimal cardioinhibition during vasovagal syncope.

**Methods:**

The study included subjects with a decrease in heart rate while experiencing a complete vasovagal syncope during tilt-table testing. The subjects were classified as having marked, intermediate or minimal cardioinhibition, based on tertile values of the decrease in heart rate. Hemodynamic parameters between these groups were compared before tilt in the supine position, shortly after tilt and during cardioinhibition.

**Results:**

A total of 149 subjects with a median age of 43 (interquartile range 24–60) years were included in the study. Among the three groups with different levels of cardioinhibition, the highest heart rate was observed in subjects with marked cardioinhibition both before and shortly after tilt and at the start of cardioinhibition. The heart rate decrease in these subjects was both larger and faster compared to subjects with minimal and intermediate cardioinhibition.

**Conclusion:**

Subjects with marked cardioinhibition have both a larger and faster decrease in heart rate compared to subjects with intermediate and minimal cardioinhibition, as early as from the start of cardioinhibition. Marked cardioinhibition is related to differences in hemodynamic profiles already present well before the start of cardioinhibition.

## Introduction

Vasovagal syncope (VVS) is the most common form of reflex syncope, with an estimated lifetime prevalence of > 30% [2, 11]. Early concepts of VVS held that the decrease in blood pressure (BP) was due primarily to a parasympathetic decrease of heart rate (HR), conventionally termed cardioinhibition (CI), and a parallel diminution of vascular tone, usually termed vasodepression (VD) [1, 8, 14, 23, 25, 30]. The latter has been regarded by some researchers as being due to a release of sympathetic arteriolar vasoconstriction, resulting in a decrease in total peripheral resistance (TPR) [30], while others did not ascribe a specific mechanism to VD [1]. Later, the importance of venous pooling during the early stages of VVS, leading to a decreasing stroke volume (SV), was acknowledged [9, 10, 14, 23, 25, 29]. We recently suggested a division of VD into ‘arterial VD,’ reflected by low TPR, and ‘venous VD,’ reflected by low SV [25–27].

The relative contributions of VD and CI in VVS vary among subjects [9]. Results from tilt table test (TTT) studies have suggested a higher incidence of asystole in younger subjects [19, 20, 28], although in some studies this finding may have been biased by early termination of the procedure by tilting back in older subjects [4, 16, 19, 22]. The modified VAsovagal Syncope International Study (VASIS) criteria defined CI as a HR < 40/min for at least 10 s or asystole > 3 s. [1]

We previously proposed a novel approach to define CI regardless of absolute HR. In this definition, CI is defined as a sustained HR decrease starting abruptly in the minutes before syncope and usually ending with syncope or termination of the test with tilt down [25]. The rationale underlying this definition is based on both a theoretical and a practical consideration. First, the HR decrease in the context of the decreasing BP in VVS indicates failure of normal baroreflex action [13, 15], thereby constituting a fundamental change in circulatory control. Second, even a modest decrease of HR at the start of CI was found to be associated with an immediate further decrease of BP, showing its relevance for maintaining BP and thereby cerebral perfusion [25]. Using our novel definition, we found that CI is a near-universal feature of VVS, with a large variation in HR decrease [25].

The aim of the present study was to further assess the impact of CI by comparing subjects with marked, intermediate and minimal CI based on CI magnitude at duration, speed and the occurrence and duration of an asystolic pause in HR. In addition, we assessed whether the amount of CI was related to hemodynamic parameters, both during CI, at the start of CI and well before the start of CI, i.e. in supine and tilted positions.

## Methods

### Inclusion criteria

Subjects and data acquisition have been detailed previously [25]. In short, patients were included from the syncope unit of the Leiden University Medical Centre. We included subjects with a history of probable VVS [2] and those with a history of provoked VVS during TTT, with or without sublingual nitroglycerin. TTT was performed with recording of continuous finger BP (Finometer [Finapres Medical Systems., Enschede, The Netherlands] or Nexfin [BMEYE, Amsterdam, The Netherlands]), at least one electrocardiography (ECG) channel, electroencephalogram (EEG) and video recording [24].

BP and HR were measured and beat-to-beat estimates of SV and TPR were derived using Modelflow (Finapres Medical Systems). Cases with presyncope only, i.e. without video- and EEG-documented loss of consciousness during TTT, or those with additional tilt-related diagnoses were excluded.

### Description of cardioinhibition

**Fig. 1:**
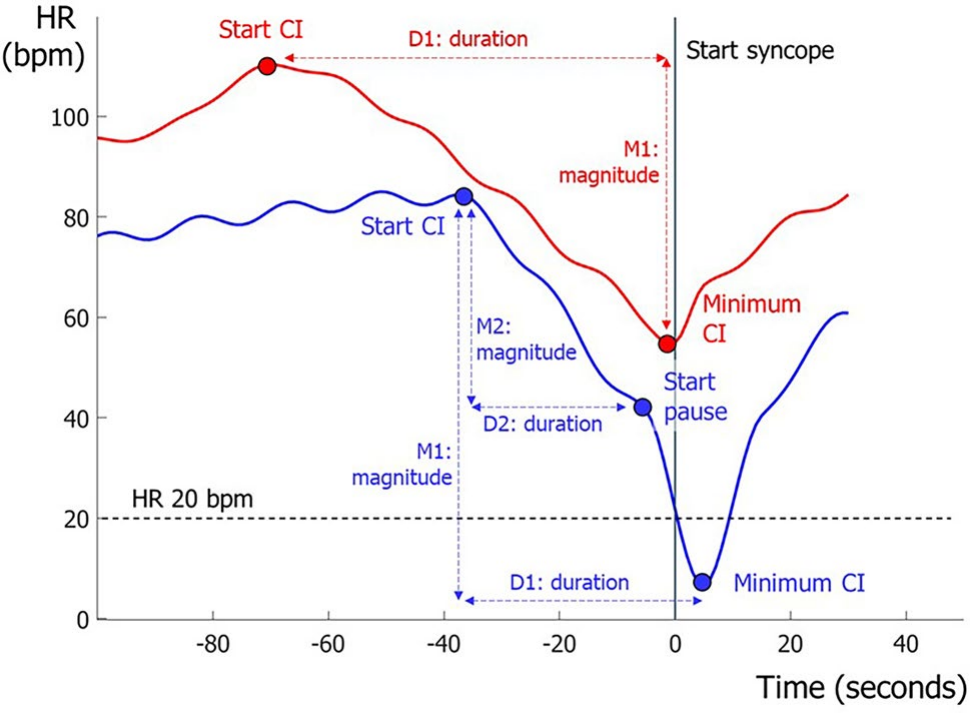
Definition of cardioinhibition. Two examples of definitions of cardioinhibition (*CI*) and asystole in two different subjects are shown. For all subjects, duration (*D1*) CI was defined as time from the start of the decrease in heart rate (*HR*) until minimum HR and magnitude CI (*M1*), namely: (HR at the start of HR decrease) — (the minimum HR ([M1]). In subjects with asystole, we defined the magnitude and duration of CI in a second way, namely as ‘duration2’ (*D2*), being from the start of HR decrease until the start of a HR pause ≥ 3 s, and ‘magnitude2’ (*M2*), namely: (HR at the start of the HR decrease) — (HR at the start of a HR pause ≥ 3 s)

We measured the time of the start of CI (Fig. 1) as the onset of a sustained progressive decrease in HR in the minutes before syncope as follows. Four of the authors experienced with TTT (JGvD, IvR, FK, MG) were shown individual HR data without recourse to BP or clinical data, as previously described [25]. The examiners decided whether a HR decrease was present and, if so, ascertained HR at CI onset and measured the onset of CI as time prior to syncope. The beat indicating ‘minimum HR’ in the period around syncope was chosen using a consensus procedure (IvR, JGvD). The difference between HR at CI onset and minimum HR was the ‘magnitude of CI’ in beats per minute (bpm), and the time elapsed between the two events was the ‘duration of CI’ in seconds (Fig. 1). Dividing magnitude by duration yielded the ‘mean velocity of CI,’ i.e. the rate of decrease of HR, in bpm per second. We assessed presence and duration of asystolic pauses in HR ≥ 3 s. Because the minimum HR was strongly influenced by the presence of an asystolic HR pause, we also investigated CI without such pauses. To do so, we calculated ‘magnitude2,’ ‘duration2’ and ‘velocity2’ by using a shorter CI period, ending at the onset of the pause, if present (Fig. 1); if no pause was present, these parameters were the same as those in the original calculation.

### Hemodynamic profiles in subjects with marked, intermediate and minimal CI

We divided subjects with CI into three subgroups with marked, intermediate or minimal CI, respectively, using tertile values of the magnitude of decrease in HR. We investigated whether these subgroups differed in duration and velocity of CI, in the occurrence of pauses ≥ 3 s and in the duration of these pauses. For each subgroup we assessed BP, HR, SV and TPR at the start of CI. In addition, we determined resting tilted and supine values for each variable. For supine values we used the mean data from − 240 s until − 20 s before the start of the head-up tilt. We similarly established mean values for the early tilted position using the period + 20 s to + 240 s after the start of the tilt up period.

### Statistical analysis

Statistical tests were performed using Matlab (ver. R2019b; The MathWorks, Natick, MA, USA).

For continuous variables, normality of distribution was assessed for each of the three subgroups with the Kolmogorov–Smirnov test. Since some distributions were skewed, we used nonparametric methods for all our analyses. Accordingly, data are described with medians, ranges and interquartile ranges (IQR). Differences were investigated using the Mann–Whitney *U*-test and Kruskal–Wallis test for quantitative data and the Chi-square test for count data.

We investigated whether a relation between severity of CI and supine HR was due to age. To do so, we used the characteristics of the line of best fit between supine HR and age of subject to calculate residuals of supine HR, devoid of age influences. We then investigated whether the residuals differed between groups of CI severity using the Kruskal–Wallis test.

We applied the Bonferroni correction for multiple testing which resulted in a threshold for significance of *p* < 0.002.

### Ethical considerations

The Medical Ethics Committee approved the protocol. According to the law of The Netherlands at the time of this study, the use of anonymous data gathered exclusively for patient care, as was the case here, did not require individual informed consent.

## Results

### Description of subjects

**Fig. 2:**
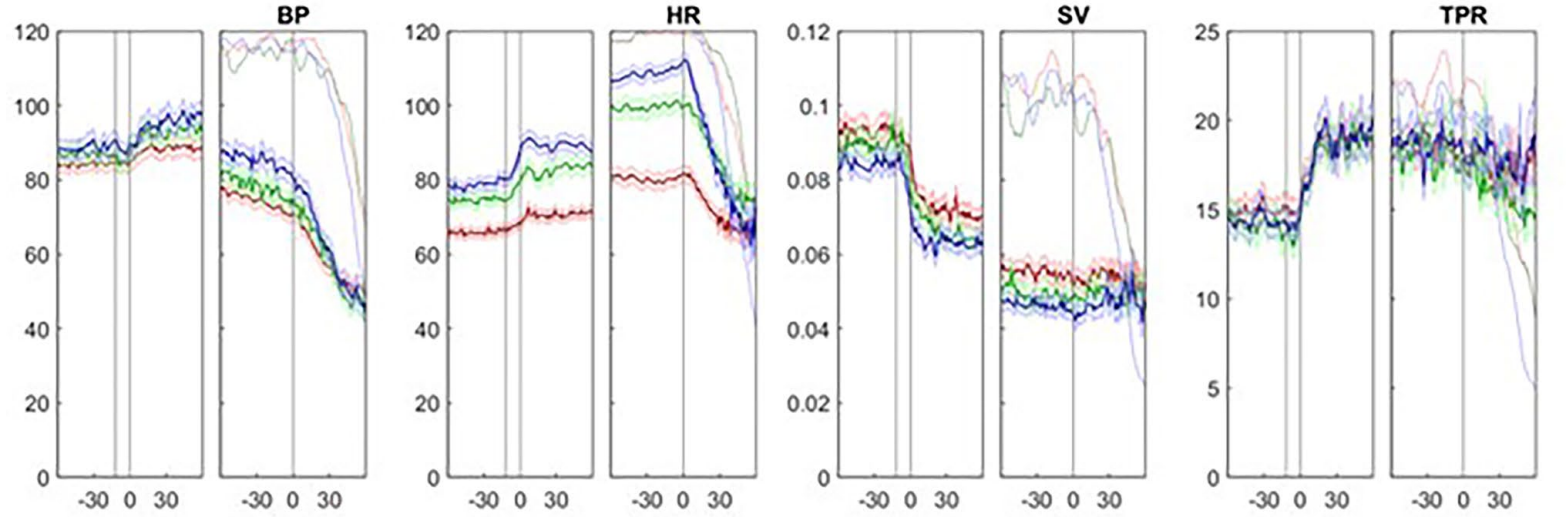
Hemodynamic parameters of 149 subjects with tilt-induced vasovagal syncope with marked, minimal and intermediate cardioinhibition, respectively. Subgroups were based on tertiles of the magnitude of cardioinhibition (*CI*). For each variable, the left panel represents the period around tilting in seconds, with negative timepoints reflecting the supine position. The right panels show the period around the start of CI, with the timepoint zero being the start of CI. The blue lines represent subjects with marked CI, the green lines are the subjects with intermediate CI and the red lines are subjects with minimal CI. The thin lines show the number of measurements for each point in time, as a percentage (with 100% at the top of the right axis).* BP* Blood pressure,* HR* heart rate,* SV* stroke volume,* TPR* total peripheral resistance

We included 149 subjects with a complete VVS during TTT and cardioinhibition (CI). The median age of the study group was 43 (IQR 24–60) years, and 44% were men. CI started a median of 58 s before syncope (range 200–12 s). The median decrease in HR was 65 (range 16–139) bpm with a median rate of HR decrease of 1 (range 0.1–5.1) bpm/s (Table 1; Fig. 2). Of 149 subjects, 68 (46%) had a pause in HR ≥ 3 s. The pauses started 51 (IQR 35–75) s after the start of CI and 5 (IQR 2–7) s before syncope. Median HR at the start of the pause was 43 (IQR 31–49) bpm. Median mean arterial pressure (MAP) at the start of the pause was 37 (IQR 28–43) mmHg. We had limited data for SV (20 subjects) and TPR (15 subjects) at the start of a pause ≥ 3 s; median SV was then 45 (IQR 32–74) ml and median TPR was 17 (IQR 15–24) mmHg min/L.

**Table 1:** Description of all subjects with complete vasovagal syncope during tilt-table test and cardioinhibition

Descriptive variables	All CI
*N*	149
Age, years	43 (24–60)
Male, *N* (%)	66 (44%)
Nitroglycerin, *N* (%)	99 (66%)
HR at start CI (bpm)	97 (83–113)
MAP at start CI (mmHg)	73(65–82)
SV at start CI (ml)	46 (37–57)
TPR at start CI (mmHg min/l)	17.0 (13.6–20.3)
Time from start CI till syncope	58 (45–83)
Minimum HR	35 (17–47)
Duration CI (s)	61 (46–84)
Magnitude CI (HR decrease, bpm)	68 (48–85)
Speed CI (HR decrease in bpm/s)	1 (0.7–1.6)
Duration HR pause if present (s)	6 (3–37)

Values arem reported as the median with 25–75 percentiles (interquartile rate) in parentheses except for numbers of patients (*N*, %)

*bpm* Beats per minute, *CI* cardioinhibition, *Duration CI* time from start CI till minimum HR, *HR* heart rate, *Magnitude CI* difference between HR at start CI and minimum HR, *MAP* mean arterial pressure, *Speed CI* magnitude CI divided by duration CI, *SV* stroke volume, *TPR* total peripheral resistance

### Hemodynamic profiles in subjects with marked, intermediate and minimal CI, respectively

The three subgroups with marked, intermediate and minimal CI differed in age and sex, with subjects with marked CI being younger and more often women than subjects with minimal CI (Table 2). The velocity of CI was higher in subjects with marked CI (*p* < 0.001).

**Table 2:** Hemodynamic parameters of subjects with vasovagal syncope with marked, minimal and intermediate cardioinhibition, respectively

	Marked CI	Intermediate CI	Minimal CI	*p* value
*N*	50	52	47	
Age (years)	34 (19–45)	42 (24–60)	51 (36–66)	< 0.001*
Male, *N* (%)	16 (32)	21 (40)	29 (62)	0.01
Asystole, *N* (%)	42 (84)	20 (38)	6 (13)	< 0.001*
Nitroglycerin, *N* (%)	38 (76)	36 (69)	25 (53)	0.05
Time until syncope (s)	534 (306–720)	606 (323–836)	612 (343–870)	0.54
Minimum HR (bpm)	10 (6–17)	32 (17–48)	44 (33–57)	< 0.001*
Magnitude CI (bpm)	98 (87–107)	66 (58–74)	38 (29–47)	< 0.001*
Duration CI (s)	62 (39–85)	74 (48–89)	78 (50–88)	0.096
Speed CI (bpm/s)	1.9 (1.2–2.4)	1.1 (0.7–1.3)	0.6 (0.3–0.9)	< 0.001*
CI parameters until start asystole				
Magnitude2	70 (58–82)	56 (46–64)	35 (27–44)	< 0.001*
Duration2	53 (34–77)	63 (46–86)	63 (47–88)	0.024
Speed2	1.5 (1.0 – 2.0)	0.79 (0.6–1.2)	0.5 (0.3–0.8)	< 0.001*
hemodynamic at start CI				
HR at start CI (bpm)	111 (100–121)	98 (84–114)	82 (79–93)	< 0.001*
MAP at start CI (mmHg)	81 (69–89)	75 (67–82)	71 (65–79)	0.02
SV at start CI (ml)	44 (33–54)	49 (34–61)	54 (41–68)	0.02
TPR at start CI (mmHg min/L)	18 (13–22)	18 (13–20)	18 (14–20)	0.93
Hemodynamic supine				
HR supine (bpm)	74 (66–82)	73 (65–79)	64 (57–70)	< 0.001*
MAP supine (mmHg)	86 (75–95)	86 (75–93)	80 (71–89)	0.2
SV supine (ml)	83 (72–98)	89 (80–103)	94 (78–108)	0.02
TPR supine (mmHg min/L)	15 (12–18)	14 (11–16)	15 (11–18)	0.6
Hemodynamic after tilt				
HR after tilt (bpm)	87 (79–95)	84 (74–92)	73 (65–82)	< 0.001*
MAP after tilt (mmHg)	95 (82–103)	94 (84–100)	89 (79–101)	0.2
SV after tilt (L)	62 (53–71)	67 (58–80)	71 (60–81)	0.02
TPR after tilt (mmHg min/L)	19 (14–22)	18 (14–20)	18 (14–22)	0.7

Values are reported as the median with 25–75 percentiles (interquartile rate) in parentheses. Group comparisons were made using Mann–Whitney and Chi-square test when applicable

*Significant difference at *p* = 0.002 after Bonferroni correction

*Duration2* Time from start CI till start of HR pause, *Magnitude2* difference between HR at start CI and HR at start of HR pause. *Speed2* Magnitude2 divided by Duration2

**Fig. 3:**
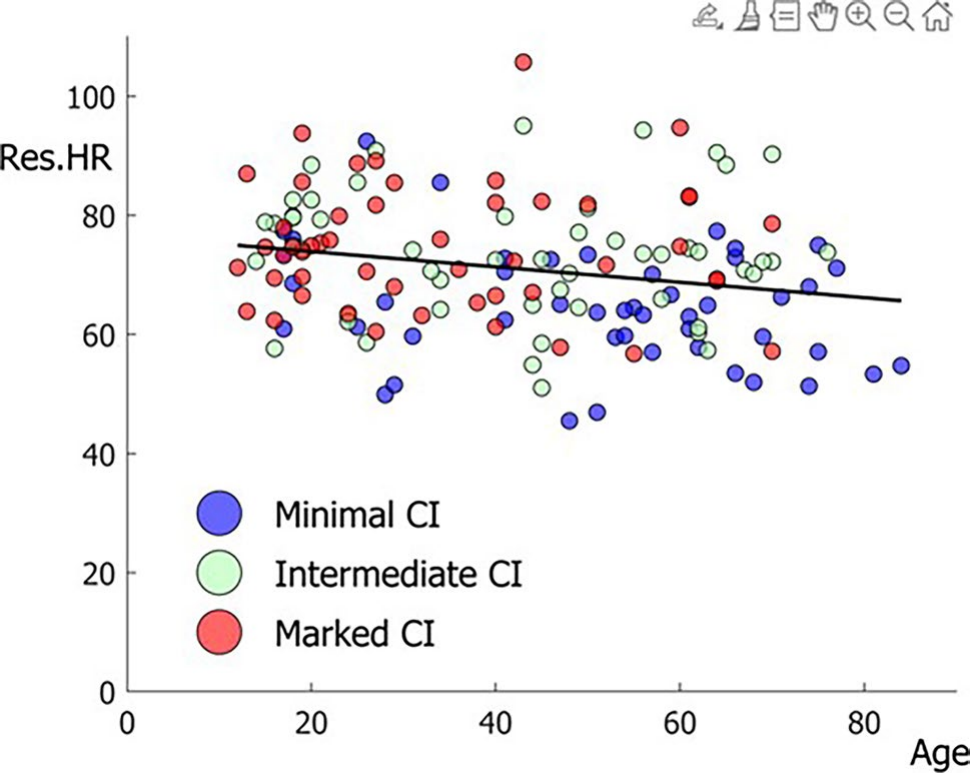
Relation between age and supine heart rate (*HR*). The relation between supine HR before tilt-up is shown as a function of age. The black line shows the line of best fit used to calculate residuals. The relation was significant (*p*  = 0.0056) albeit with limited explained variance (*R*^2^ = 0.05). The three subgroups of cardioinhibition (*CI*) severity are indicated. Note that the age ranges of the groups overlap, and that supine HR was highest for those with marked CI (red markers)

The magnitude of CI was inversely related to median HR at the start of CI; subjects with marked CI had the highest HR at the start of CI, while subjects with minimal CI had the lowest HR at the start of CI, with those with intermediate CI falling in between. This difference in HR between the three subgroups was not restricted to the start of CI, as subjects with marked CI also had a higher baseline HR, both in the supine and in the tilted position. SV tended to be lower in subjects with marked CI, both at the start of CI and in the supine position and tilted position, although the difference was not statistically significant. HR increased in the minutes before the start of CI only in subjects with marked CI (Table 2; Fig. 3).

Supine HR was linearly related to age (Fig. 3; *p* = 0.006) with HR decreasing by only 1.3 bpm per decade. Residuals differed between groups of CI severity (Kruskal–Wallis, *p* < 0.00001); post-hoc tests showed differences between marked and minimal CI (*p* < 0.0001), between intermediate and minimal CI (*p* = 0.0002), but not between marked and intermediate CI (*p* = 0.87).

The occurrence of HR pauses was associated with CI magnitude: an asystolic pause of ≥ 3 s occurred in 84% of subjects with marked CI, 38% of those with intermediate CI and 13% in subjects with minimal CI (*p* < 0.001). The duration of a pause did not differ significantly between the three subgroups, although median duration increased in the order marked, from intermediate to minimal CI. Analysis of CI without the influence of pauses (Table 2) showed that magnitude2 and velocity2, but not duration2, differed significantly between the three subgroups, in the sense that both magnitude and velocity were larger in subjects with marked CI than in those with minimal CI and intermediate CI in between; these results showed that pauses were not the sole expression of differences in severity of CI.

## Discussion

This study of CI in subjects with TTT-induced VVS revealed two novel findings. First, subjects with marked CI had a different course of CI, with a faster decrease in HR and a higher frequency, but not a longer duration of an asystolic pause, compared to subjects with minimal CI. Second, subjects with marked CI had a different hemodynamic profile, with a higher HR, compared to subjects with minimal CI, that was already present in the supine position, well before the start of CI.

### Cardioinhibition in VVS

The percentage of subjects with CI we reported in this study was higher than that in some other tilt-table studies, possibly due in part due to our novel definition of CI, according to which small decreases in HR are still recognized as CI. In addition, 42% of our subjects had an asystolic pause in HR ≥ 3 s, which is higher than the 6–32% described in the literature [4]. Our high rate of pause in HR is probably due to our strict inclusion criteria, with only subjects with complete syncope documented with video EEG, as the occurrence of CI and asystole during a TTT strongly depends on decisions when to tilt back [20, 28].

HR showed pronounced interindividual variability at the start of an HR pause ≥ 3 s, indicating that it is not a low HR itself that induced a pause. In those with a pause, SV just prior to the start of asystole was similar to SV at the start of CI. Although this result is hampered by limited data, it pleads against the ‘empty heart theory’ as a trigger for a pause in HR [18].

### Hemodynamic profiles in subjects with marked, intermediate and minimal CI

Although all subjects had CI, the degree of CI, reflected by CI magnitude (i.e. HR decrease) and speed of CI, varied considerably. CI magnitude was associated with both age and gender; the group with marked CI was younger and consisted of more women than the group with weak CI, with the intermediate group lying in between; this result is in line with previous studies on CI related to age [16, 19, 20, 22]. A novel finding was that subjects with marked CI had higher HR and lower SV, not only at the start of CI, but already in the supine position before upright tilt. This relation between severity of CI and supine HR was not explained through effects of age, as supine HR decreased only with 1.3 bpm/decade and subjects with marked HR had the highest supine HR at all ages.

We defined subgroups based on HR decrease, so by definition subjects with marked CI had the largest CI magnitude. An interesting new finding was that the speed of HR decrease was also higher in subjects with marked CI, as early as from the start of CI and regardless of the occurrence of a pause in HR.

A novel finding regarding HR pauses was that the duration of a pause did not depend on CI magnitude, although a pause occurred more frequently in those with marked CI.

In subjects with marked CI, we noted a short increase in HR just before the start of CI, which was not present in subjects with weak or intermediate CI. At this point in time, just before the start of CI, BP was already gradually decreasing in all subgroups. The final increase in HR in the strong CI subgroup can be seen as a last-ditch physiological attempt to increase BP. This HR increase in the strong CI subgroup, which contained younger subjects, is in line with the previous finding that BP regulation depends stronger on HR in the young and more on TPR in the elderly [28].

### Limitations

There are a number of limitations to this study. First, we studied tilt-induced VVS, which means that hemodynamic changes might differ from those in spontaneous VVS. There is some evidence that bradycardia might be more prominent in ‘real-life’ VVS compared to tilt-induced VVS, so we may have underestimated CI magnitude or speed [17]. Second, while we studied a large number of subjects (*N* = 149) with complete VVS and CI, some data were missing around syncope, especially for SV and TPR. Third, the division according to magnitude of CI was linked to differences in age and sex between the groups. We did not attempt to correct for sex while evaluating CI severity. However, as sex is known to affect both CI in VVS and hemodynamic parameters, this might have affected our results [5, 7, 12, 19, 28]. Fourth, we used Modelflow, which estimates SV and subsequently CO and TPR from BP waveform, instead of direct intra-arterial measurement or thermodilution measurement. Modelflow is known to have some limitations in terms of accuracy of absolute values of CO and related variables, especially when mean arterial pressure drops < 60 mmHg and when large abrupt TPR changes occur [3, 6, 21]. However, as Modelflow has been shown to reliably measure relative changes in hemodynamic parameters, we believe that the effect of any Modelflow inaccuracy on the present findings is limited, as we also described in more detail previously [25].

### Clinical implications

We have identified two features that may help identify subjects with marked CI in an early stage: first, such subjects had a higher HR already in the supine position; and, second, their HR declined faster from the start of CI.

## Conclusion

Subjects with marked, intermediate and minimal CI had a different course of CI and different hemodynamic profiles both during CI and before the start of CI, as early as in the supine position. Our findings help to further understand the occurrence and differences of cardioinhibition in VVS and may aid to improving treatment strategies.
